# Prospective observational study investigating the factors impacting the safety of endoscopic right-sided colectomy for colon cancer in Japan (SCaRLET study): a study protocol

**DOI:** 10.1186/s12893-025-03362-1

**Published:** 2025-11-29

**Authors:** Nobuki Ichikawa, Daisuke Yamamoto, Yukitoshi Todate, Ken Imaizumi, Takuya Miura, Akihiro Kondo, Koya Hida, Tomonori Akagi, Tomohiro Yamaguchi, Shigenori Homma, Akinobu Taketomi, Seiichiro Yamamoto, Kay Uehara, Koji Okabayashi, Hiroaki Iijima, Tsukasa Akahane, Takeshi Naitoh, Ichiro Takemasa, Shigeki Yamaguchi, the SCaRLET study group collaboratives in the Japan Society of Laparoscopic Colorectal Surgery

**Affiliations:** 1 Department of Gastroenterological Surgery 1, Hokkaido University Graduate School of Medicine, Sapporo, Japan; 2 Department of Gastrointestinal Surgery/Breast Surgery, Kanazawa University, Kanazawa, Japan; 3 Department of Surgery, Southern Tohoku Research Institute for Neuroscience, Southern Tohoku General Hospital, Koriyama, Japan; 4 Department of Gastroenterological Surgery, Hirosaki University Hospital, Hirosaki, Japan; 5 Department of Gastroenterological Surgery, Faculty of Medicine, Kagawa University, Kagawa, Japan; 6 Department of Surgery, Kyoto University Hospital, Kyoto, Japan; 7 Department of Gastroenterological and Pediatric Surgery, Oita University, Oita, Japan; 8 Department of Gastroenterological Surgery, Cancer Institute Hospital of Japanese Foundation for Cancer Research, Tokyo, Japan; 9 Department of Gastroenterological Surgery, Tokai University School of Medicine, Isehara, Japan; 10 Department of Gastroenterological Surgery, Nippon Medical School, Tokyo, Japan; 11 Department of Surgery, Keio University School of Medicine, Tokyo, Japan; 12 Department of Lower Gastrointestinal Surgery, Kitasato University School of Medicine, Sagamihara, Japan; 13 Department of Gastroenterological Surgery, Osaka International Medical and Science Center, Osaka Keisatsu Hospital, Osaka, Japan; 14 Division of Colorectal Surgery, Department of Surgery, Tokyo Women’s Medical University, 8-1 Kawada-cho, Shinjuku-ku,Tokyo, 162-8666 Japan

**Keywords:** Colorectal cancer surgery, Laparoscopic colorectal surgery, Complications, Robotic surgery, Vascular injury

## Abstract

**Background:**

Right-sided colon resection is generally regarded as a relatively safe laparoscopic procedure with a low complication rate. Paradoxically, right hemicolectomy has been reported to carry a fourfold higher mortality rate compared to low anterior resection for rectal cancer in Japanese real-world data, despite the latter being more technically demanding and associated with a higher complication rate. This study aims to investigate the factors that influence the safety of laparoscopic right-sided colon resection.

**Methods:**

Patients undergoing laparoscopic or robot-assisted surgery for right-sided colon cancer at participating institutions in the Laparoscopic Colorectal Resection Study Group will be prospectively enrolled between 3 July 2024 and 31 May 2026. This multicenter study expects to enroll a total of 2,000 colorectal cancer cases from 73 facilities nationwide. The primary endpoint is the incidence of postoperative complications classified as Clavien–Dindo grade ≥ III. Risk factors contributing to these severe complications will be analysed. As a key secondary endpoint, the incidence and predictors of intraoperative vascular injury near the superior mesenteric vein will also be evaluated.

**Discussion:**

This nationwide, multicentre, prospective study (SCaRLET study) will provide critical insights into the safety of laparoscopic and robot-assisted right-sided colectomy. By evaluating 2,000 cases under standardised conditions, the study aims to clarify the actual incidence and causes of severe postoperative complications and intraoperative vascular injuries, which remain poorly understood despite the procedure’s perceived safety. Comprehensive data collection and adherence to routine clinical practice will support accurate risk assessment and enhance the generalizability of findings. The results are expected to inform best practices and contribute to improving surgical safety in colorectal procedures including indication of colectomy both in Japan and internationally.

**Trial registration:**

This study was registered in the Japan Registry of Clinical Trials on 3 July 2024 (jRCT1010240021).

**Supplementary Information:**

The online version contains supplementary material available at 10.1186/s12893-025-03362-1.

## Background

Laparoscopic surgery for colorectal cancer is becoming more common worldwide and has demonstrated superiority to open surgery regarding short-term outcomes [1–3] and non-inferiority regarding long-term outcomes [4–6]. Among laparoscopic colorectal resections, right-sided colectomy is a relatively safe procedure, with few complications. An annual report of a Japanese nationwide survey in 2020 indicated that right hemicolectomy had a lower complication rate (8.0%) compared to low anterior rectal resection (11.1%) [7]. However, right hemicolectomy had a high 30-day mortality rate of 1.4%, four times more than that of low anterior rectal resection, which is a more difficult procedure with a higher complication rate [7]. More than 30% of patients undergoing right hemicolectomy are older than 80 years, which is more than twice as many as those undergoing low anterior rectal resection [8]. This older population might include many high-risk patients, and surgery might be indicated regardless. One hypothesis is that postoperative mortality following right hemicolectomy is primarily influenced by non-operative factors such as advanced age and frailty. However, operative factors — including intraoperative complications and delayed recognition of clinical deterioration — may also play a role. In other words, a single complication may present more severely, with a delayed response exacerbating severity. Therefore, further scientific investigation is required to clarify the factors affecting severe complications, including mortality, after laparoscopic right-sided colectomy.

Regarding procedural issues, intraoperative vascular injury remains a major complication of this technique. Injury to the superior mesenteric vein (SMV) and surrounding vasculature can cause major bleeding and subsequent massive bowel resection. Approximately 3% of D3 dissections result in vascular injury [8]. Moreover, minor injuries that do not result in major bleeding may be encountered clinically; however, they do not significantly affect final outcomes. Nevertheless, clinicians, particularly young surgeons, should be aware of these risks to safely perform the procedure. Clarifying the frequency and risk factors of intraoperative vascular injuries is crucial. Therefore, we designed the current prospective observational study to investigate factors affecting the intraoperative vascular injury rate as additional safety indicators.

**Fig. 1:**
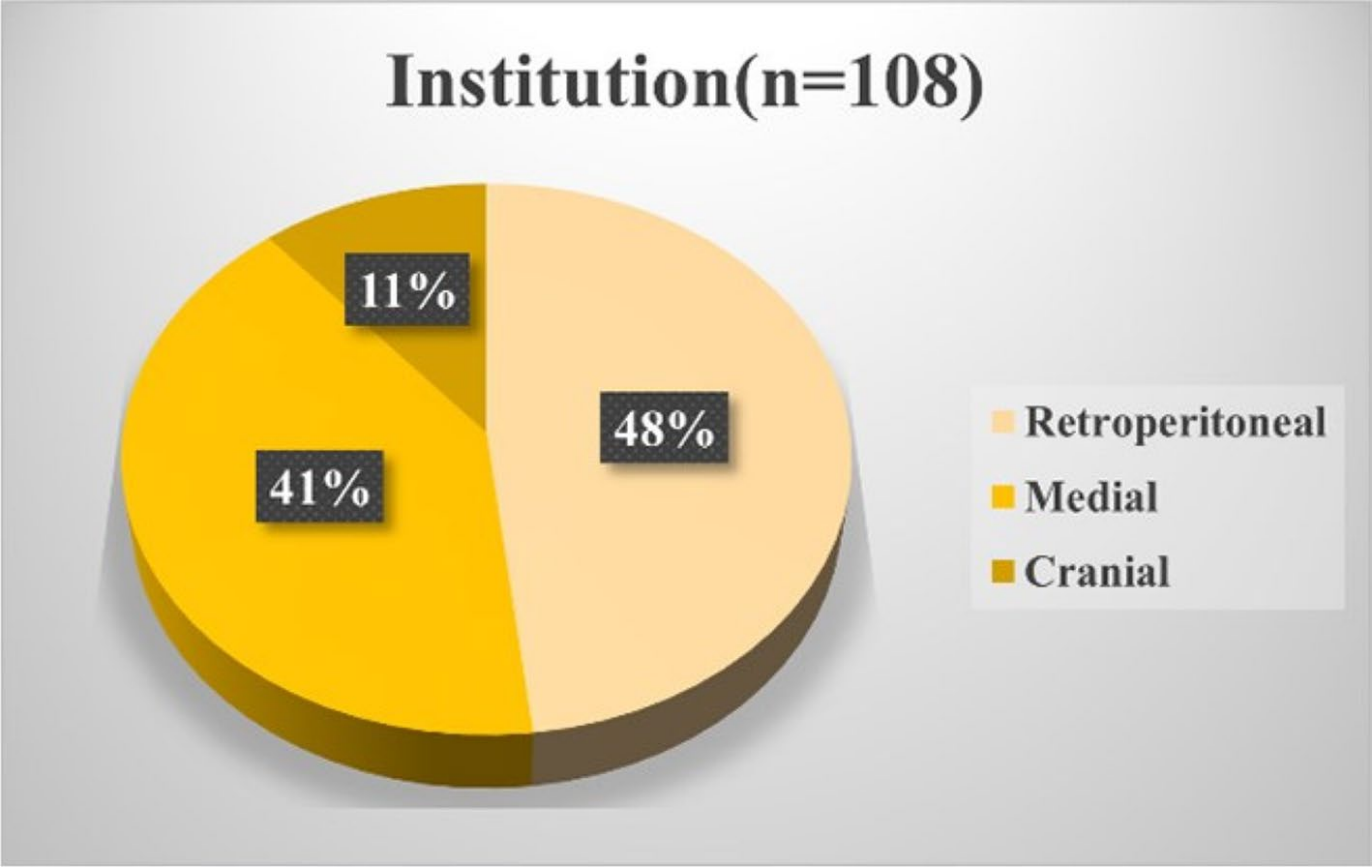
Questionnaire regarding the preferred approach for conventional laparoscopic right colectomy**.** A nationwide questionnaire was conducted in 2023 by the Japan Society of Laparoscopic Colorectal Surgery. The most preferred approach in each institution is shown. According to the survey, the medial and retroperitoneal approaches are the most preferred

**Fig. 2:**
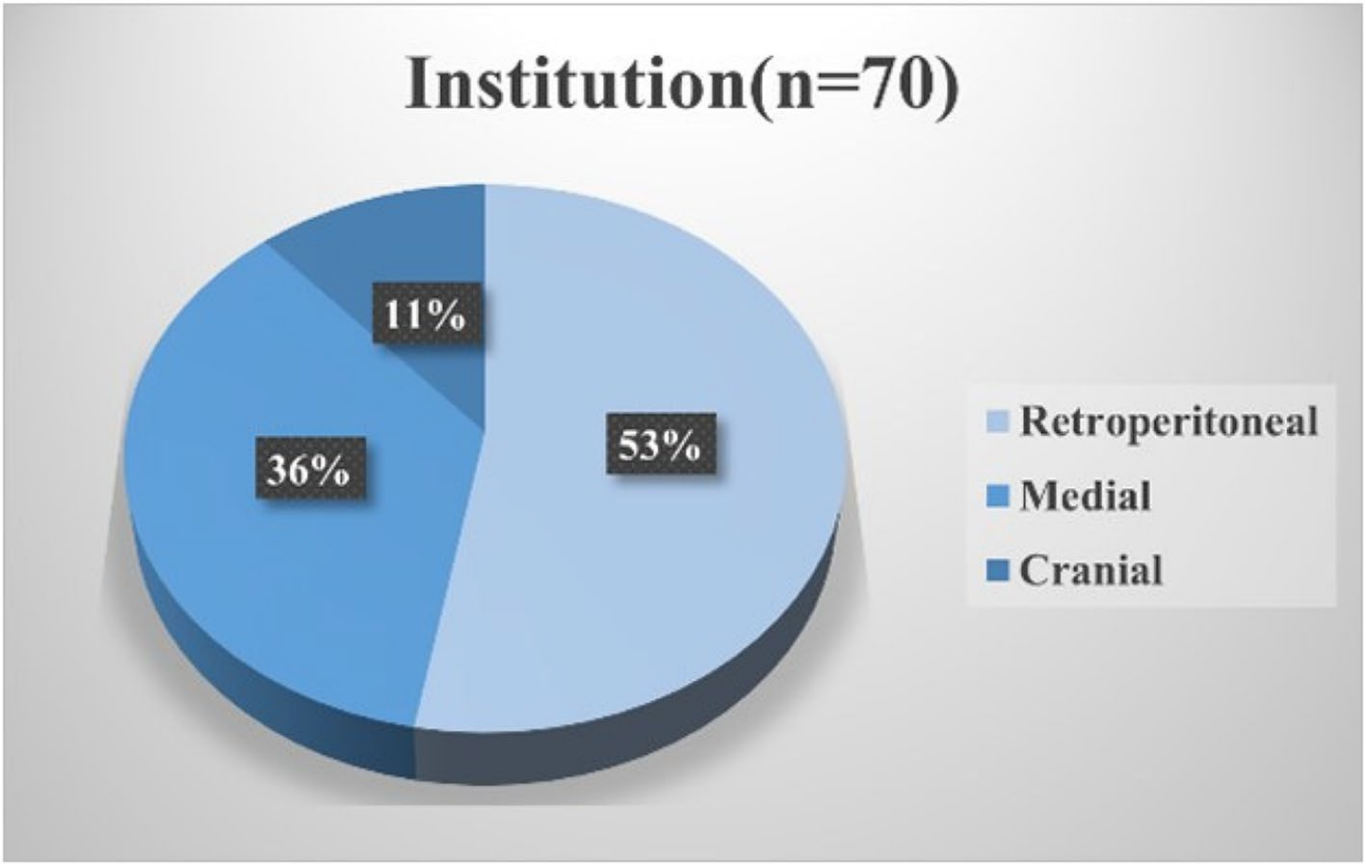
Questionnaire regarding the preferred approach for robot-assisted right colectomy. A nationwide questionnaire was conducted in 2023 by the Japan Society of Laparoscopic Colorectal Surgery. The most preferred approach in each institution is shown. According to the survey, the medial and retroperitoneal approaches are the most preferred

**Fig. 3:**
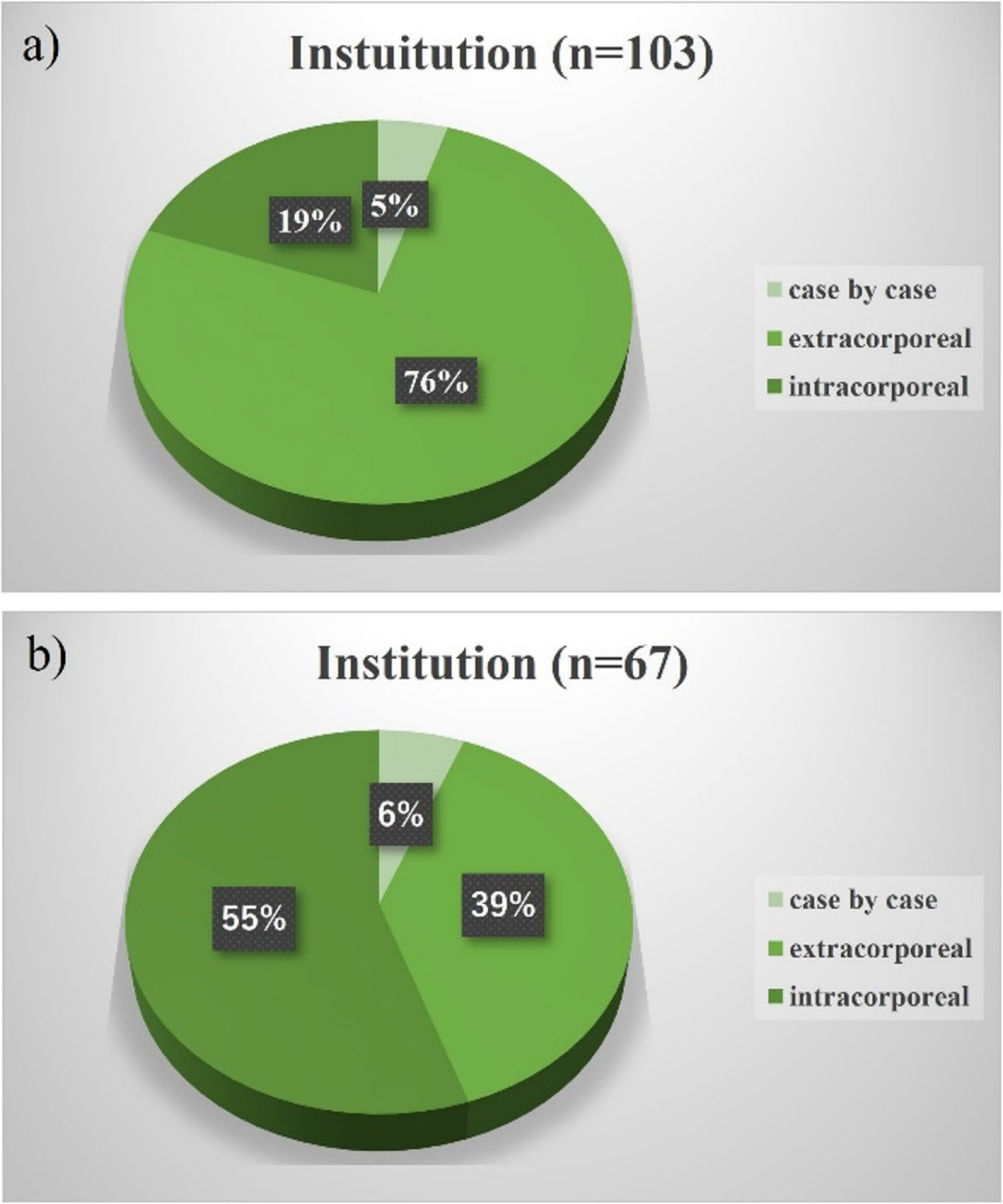
Questionnaire regarding the preferred anastomosis for laparoscopic right colectomy. A nationwide questionnaire conducted in 2023 by the Japan Society of Laparoscopic Colorectal Surgery revealed institutional preferences for anastomosis techniques. (**a**) Extracorporeal anastomosis was predominant in conventional laparoscopic surgery, (**b**) while intracorporeal anastomosis was favoured in robot-assisted procedures

Moreover, several concerns regarding right-sided colectomies require resolution. First, right-sided colectomy involves complete mesenteric excision (CME; in which regional lymph nodes in the mesentery are completely removed) and central vascular ligation (CVL; in which the dominant vessels in the mesentery at the trunk are removed) [9]. CME with CVL is technically feasible [8, 10, 11] and oncologically beneficial [12–14]. However, the interpretation of the ‘trunk’ is controversial in Japan [15]. It remains inconclusive whether the left margin of the anterior aspect of the SMV (D3v) or the superior mesenteric artery (SMA) (D3a) is appropriate (technical safety and oncological benefit) for the most central margin of lymph node dissection, along with CVL. Second, it remains unclear which surgical technique is the safest among the medial approach [16–18], the cranial approach [19], and approaches preceded by retroperitoneal dissection [20, 21]. A survey conducted by the Japan Society of Laparoscopic Colorectal Surgery (JSLCS) demonstrated that the medial and retroperitoneal approaches were the two main approaches (Figs. 1 and 2). However, few reports confirm which is the safest procedure [22–24]. Moreover, outcome superiority between robotic and laparoscopic surgery for right hemicolectomy remains inconclusive [25–28]. Third, the surgeon’s proficiency may play an important role in safety. The participation of surgeons certified through the Endoscopic Surgical Skill Qualification System, as defined by the Japanese Society for Endoscopic Surgery, has been shown to contribute to ensuring surgical safety in rectal cancer procedures [29, 30]; however, this has not been clarified in laparoscopic right-sided colectomy. Moreover, the safest surgical technique (medial or retroperitoneal approach, robotic or not) to be performed by young surgeons (e.g., before being qualified as a board-certified gastrointestinal surgeon) remains to be determined. Finally, the safest anastomotic method remains unclear (Fig. 3), with the most significant issue being whether intracorporeal or extracorporeal anastomosis is more useful [31–34]. In the current study, we structured the data collection to address these unresolved issues through a large-scale prospective cohort.

Given the above background, we have designed this prospective observational study to investigate factors influencing the safety of laparoscopic right-sided colon resection.

## Methods

### Eligibility criteria

Patients scheduled to undergo laparoscopic surgery for right-sided colon cancer between 3 July 2024 and 31 May 2026 at a facility affiliated with the JSLCS will be eligible for this study.

### Selection criteria

1. Cases with a diagnosis of adenocarcinoma.
2. Cases in which laparoscopic ileocecal resection, laparoscopic right hemicolectomy, or laparoscopic extended right hemicolectomy with D2 or D3 dissection are planned for clinical stage 0, I, II, or III colon cancer in the caecum, ascending, or transverse colon.
3. Robot-assisted surgical cases will be eligible for inclusion only after the participating institution has demonstrated procedural proficiency through completion of at least 10 prior cases of ileocecal resection, right hemicolectomy, or extended right hemicolectomy for right-sided colon cancer.

### Exclusion criteria

1. Multiple colorectal cancers or multiple other primary malignancies (concurrent multiple cancers with a disease-free interval of ≤ 5 years at the time of registration). However, lesions equivalent to intraepithelial or intramucosal carcinomas, which are considered curable with local treatment, will not be considered as multiple cancers.
2. Ulcerative colitis-related cancers.
3. Cases in which patients request to withdraw from participation in this study.
4. Cases deemed inappropriate by the investigator.

### Endpoints

The primary endpoint is the incidence of postoperative complications (Clavien–Dindo classification grade ≥ III) [35]. The rate of severe postoperative complications in all eligible patients will be investigated, and risk factors affecting them will be determined.

A key secondary endpoint is the incidence of intraoperative vascular injury around the SMV. The rate of intraoperative vascular injury around the surgical trunk in all eligible patients will be investigated, and risk factors affecting it will be extracted.

Other secondary endpoints include the incidence of postoperative complications (Clavien–Dindo classification grade ≥ II), 5-year overall survival, recurrence-free survival, and other short- and long-term outcomes (operative time, blood loss, conversion rate, degree of lymph node dissection, intraoperative complication rate, reoperation rate, R0 resection rate, local recurrence rate in the whole cohort and by stage, and incidence of bowel obstruction and herniated abdominal wall scarring). Factors influencing the above endpoints will be determined for all eligible patients. Furthermore, comparisons will be made between each subgroup, as shown in Table 1.

**Table 1:** Secondary endpoints

1. Short-term results by approach (a. Medial approach vs. retroperitoneal approach; b. Medial approach vs. cranial approach; c. Retroperitoneal approach vs. cranial approach; a–c for laparoscopic surgery and robotic surgery, respectively)
2. Short-term results of robot-assisted and laparoscopic surgery (Comparison in all approaches, medial approach, retroperitoneal approach, and in each ASA-PS, respectively)
3. Short-term results by degree of lymph node dissection (D2, D3v, D3a)
4. Long-term results by approach, robot or laparoscopy, and degree of dissection
5. Impact of experience on short-term and long-term outcomes after the surgery performed by the surgeons before obtaining board certification in gastrointestinal surgery (including the investigation by each approach, robot/laparoscopy, and degree of lymph node dissection)
6. Impact of surgeons’ case volume prior to obtaining board certification in gastrointestinal surgery on short- and long-term outcomes following robot-assisted procedures
7. Impact of the participation of Endoscopic Surgical Skill Qualification-certified surgeons in surgery and their role in the procedure on short- and long-term outcomes
8. Impact of anastomosis method on short- and long-term outcomes
9. Relationship between the presence of drain insertion and incidence and severity of complications
10. Risk factors for intestinal obstruction (long- and short-term)
11. Risk factors for complications and severity of complications in older adults

*ASA-PS* American Society of Anesthesiologists physical status

### Observation, investigation data, and methods

Data will be obtained from the participants’ medical records. All data to be obtained are used in daily medical care, and their frequency is equivalent to it. The principal investigator or co-investigator at each site is responsible for registering cases and entering medical data into the Electronic Data Capture system. Recording by a medical assistant is also permitted, provided it is approved by the principal investigator or co-investigator.

1. Participating institution background
  Institution type and size
2. Patient background
  Patient age; sex; body mass index; American Society of Anesthesiologists Physical Status; Eastern Cooperative Oncology Group performance status; albumin level; lymphocyte count; presence of dementia, diabetes, chronic kidney disease, chronic liver disease, or respiratory comorbidities; history of cardiac or cerebrovascular disease; use of steroids/immunomodulators and antithrombotic drugs; preoperative bowel obstruction; preoperative anaemia; abscess or peritonitis; preoperative therapy; history of laparotomy; timing of surgery; primary tumour location; and tumour stage at initial diagnosis (clinical stage, T factor, N factor).
3. Technique and surgeons
  Whether the surgeon is a board-certified gastrointestinal surgeon, whether an Endoscopic Surgical Skill Qualification-certified surgeon participates in the operation and his or her role, the approach, blood vessels to be dissected, procedure of dissecting the accessory right colic vein (via the cranial or caudal side), level and direction of lymph node dissection, instruments used during node dissection, method of anastomosis, combined resection of other organs, whether a robot is used, number of previous robotic-assisted right-sided colon resections (ileal resection, right hemicolectomy, or extended right hemicolectomy), and whether a robotic surgery proctor participates in the surgery if robotic surgery is used.
4. Surgical outcomes
  The date of surgery, operation time, blood loss, presence of intraoperative vascular injury, other intraoperative complications, 30-day postoperative survival, open conversion, intraoperative blood transfusion, details and grade of postoperative complications within 30 days (Clavien–Dindo classification grade ≥ II), onset and date of intervention for cases with anastomotic leakage/deep wound infection (surgical site infection [SSI]), enforcement and date of reoperation, drain placement, anti-adhesive placement, date of discharge, and operative death.
5. In cases of intraoperative vascular injury:
  Timing of intraoperative vascular injury; response to intraoperative vascular injury; unplanned bowel resection due to bleeding; open conversion due to bleeding; final post-haemostatic status; and amount of blood transfusion, blood products, and albumin administered from intraoperative to postoperative day 3.
6. Surgical deaths
  Cause of death, date of death, and date of therapeutic intervention.
7. Pathological and histological findings and postoperative course
  Tumour diameter, histological type (main), pathological stage, T-factor, N-factor, R-status, number of harvested lymph nodes, number of lymph node metastases, presence and details of adjuvant therapy, survival outcome, date of confirmation of final survival outcome, date of confirmation of recurrence, and type of initial recurrence.
  Patients with pStage 0 and pStage IV will be censored, and no follow-up will be performed after initial postoperative discharge. The development of intestinal obstruction (Clavien–Dindo classification grade ≥ II) and abdominal wall scar hernia will be investigated during follow-up. Patients will be followed up during the postoperative period according to the Japanese Society for Cancer of the Colon and Rectum guideline schedule, and tumour marker levels, thoracoabdominal computed tomography (CT) results, and colonoscopy results will be collected. Omitting observations as part of routine medical care will be deemed acceptable at the discretion of the treating physician [36].

### Definition of assessment data

1. Type of institution: distinguishes between university-affiliated hospitals or cancer centres, equivalent institutions, and others.
2. Size of institution: number of laparoscopic right-sided colectomies (ileal resection, right hemicolectomy of the colon, or right hemicolectomy of the extended colon) for colorectal cancer per year (including robotic surgery).
3. Definitions of the preoperative medical conditions are described in Table 2.

**Table 2:** Definitions of preoperative medical conditions

ASA-PS	American Society of Anesthesiologists physical status classification as described in the anaesthesia record
ECOG-PS	Score 0–4 (https://jcog.jp/doctor/tool/ps/) 0: Able to perform activities without any problems. Can perform the same daily activities as before the onset of illness without limitations 1: Physically able to walk and perform light or sedentary tasks, although physically strenuous activities are limited 2: Able to walk and perform all personal activities but not work. Spends > 50% of the day out of bed 3: Able to perform limited personal activities. Spends > 50% of the day in bed or chair 4: Unable to move at all. Unable to perform any personal activities. Spends all day in bed or chair
Dementia	Case officially diagnosed by a psychiatrist
Diabetes	On oral or insulin therapy. Or untreated and HbA1c level above the institutional norm. If HbA1c level is ≥ 8.0 or higher or equivalent within 2 weeks before surgery, it is considered poorly controlled
Chronic kidney disease	eGFR < 40. Classified as with/without dialysis
Cardiac disease	A history of myocardial infarction or angina, or heart failure presenting with fatigue, dyspnoea, or anginal symptoms during routine physical activity (equivalent to NYHA class II or higher), or more severe manifestations, is noted. Other cardiac conditions include moderate or greater aortic stenosis, arrhythmias excluding sporadic extrasystoles, post-pacemaker or ICD implantation, pulmonary hypertension, and cardiac sarcoidosis. Cases of unstable angina, myocardial infarction occurring within 7–30 days of onset, left ventricular ejection fraction (LVEF) < 40% or equivalent, and severe aortic stenosis are specifically categorised as high-risk
Cerebrovascular disease	Cerebrovascular disease will be categorised as follows: - History of cerebrovascular events with minimal or no residual deficits, including transient ischaemic attack (TIA) - Untreated cerebrovascular stenosis - Cerebrovascular disease with persistent neurological sequelae
Respiratory comorbidity	Patients with dyspnoea or more severe symptoms when walking uphill or on stairs (Hugh–Jones II equivalent). Cases on HOT therapy are included in the severe category
Asthma	Cases currently on medical therapy. This will be classified as inadequate/poorly controlled if symptomatic or with decreased forced expiratory volume% in 1 s within 1 month before surgery, or equivalent
Steroid/immunomodulator usage	Any type and dose being used at the time of initial diagnosis
Chronic liver disease	Child–Pugh B or higher, to be distinguished from Child–Pugh C
Antithrombotic drugs	Classified as none, on medication, or withdrawal. Any type of drug. If more than one drug is taken on the day of surgery, the patient is considered to be on medication

*ASA-PS* American Society of Anesthesiologists physical status, *ECOG-PS* Eastern Cooperative Oncology Group performance status, *HbA1c* glycated haemoglobin, *HOT* home oxygen therapy, *NYHA* New York Heart Association, *ICD* implantable cardioverter defibrillator, *LVEF* left ventricular ejection fraction, *TIA* transient ischaemic attack

(4) Preoperative bowel obstruction: requires intervention, such as quasi-emergency surgery, emergency surgery, preoperative gastrointestinal tube insertion, preoperative stent insertion, or stoma creation.
(5) Preoperative therapy: at least one preoperative administration of anticancer or other drugs.
(6) Laparotomy history: history of open or laparoscopic appendectomy or transabdominal preperitoneal (TAPP) hernia repair, procedures other than open or laparoscopic appendectomy or TAPP hernia repair, or none.
(7) Timing of surgery: classified as routine, semi-urgent (performed within 1 week of the initial visit or with an earlier surgical schedule between routine and urgent), or urgent (first visit or the day after; the day of or after the onset of perforation, obstruction, bloody bowel discharge, etc.).
(8) Primary tumour site: according to the Japanese Classification of Colorectal, Appendiceal, and Anal Carcinomas [37].
(9) Stage at initial diagnosis: T and N staging according to the Japanese Classification of Colorectal, Appendiceal, and Anal Carcinomas [37].
(10)  Board-certified gastrointestinal surgeon participation: Whether the surgeon is board-certified by the Japanese Society of Gastroenterological Surgery. A surgeon is classified as a complete, partial, or non-certified operator.
(11)  Robotic surgery proctor participation: in robotic surgery, participation of a proctor certified by the Japan Society for Endoscopic Surgery as a surgeon, assistant, or consultant for robotic colon or colorectal procedures.
(12) Endoscopic Surgical Skill Qualification System (ESSQS)-certified surgeon participation: whether an ESSQS-certified surgeon (certified by the Japan Society for Endoscopic Surgery) participates as a surgeon, assistant, scope operator, consultant, or partial participant (either as a partial surgeon, assistant, consultant, or scope operator).
(13)  Dissected vessels: Record whether the ileocecal artery, right colic artery, and the main trunk or branches of the middle colic artery are dissected. Also document whether accompanying veins are dissected and whether the anterior surface of the superior mesenteric artery (SMA) or superior mesenteric vein (SMV) is exposed.
(14) Approaches: classified as medial, retroperitoneal, cranial, or other.

The definitions of these approaches were compiled using a previous survey on the details of each approach used in the attending institutions [see Additional file 1]. The description below covers the details of each approach used in the institutions that responded to the survey. Broadly speaking, the medial and cranial approaches are defined as procedures in which lymph node dissection precedes mesocolic mobilisation, whereas the retroperitoneal approach is defined as one in which mesocolic mobilisation precedes lymph node dissection.

Medial approach: dissection is initiated from the caudal side of the ileocecal artery, and lymph node dissection is performed prior to mesocolic mobilisation. The basic surgical procedure includes the following: (i) dissection of the ileocecal artery and vein from the retroperitoneum; (ii) lymph node dissection of the surgical trunk; (iii) dissection of the accessory right colonic vein; and (iv) mesocolic mobilisation by dissection of the cranial, caudal, and lateral attachments. This approach also includes cases in which cranial mobilisation, which creates a cranial termination point for lymph node dissection, is preceded by the standard procedure.

Retroperitoneal approach: mobilisation of the mesocolon from the retroperitoneum is initiated at its caudal origin, and lymph node dissection is performed after mesocolic mobilisation. The basic surgical procedure includes the following: (i) mobilisation of the mesocolon from the retroperitoneum initiated at its caudal origin (regardless of the degree of lateral mobilisation); (ii) lymph node dissection of the surgical trunk; and (iii) dissection of the accessory right colonic vein, opening of the omental bursa, or hepatic mobilisation. This approach also includes cases in which cranial mobilisation, which creates a cranial termination point for lymph node dissection, is preceded by standard lymph node dissection.

Cranial approach: dissection begins by opening the omental bursa and hepatic attachment, and lymph node dissection is performed prior to mesocolic mobilisation. The basic surgical procedure includes the following: (i) opening of the omental bursa, (ii) dissection of the right accessory colic vein or hepatic attachment, (iii) lymph node dissection along the surgical trunk, and (iv) mobilisation of the lateral/caudal attachments. Whether lymph node dissection is performed from the cranial to the caudal side or from the caudal to the cranial side does not matter.

(15)  Robot usage: classified into da Vinci (except SP), Hinotori, Hugo, other models, or no usage.
(16)  Anastomosis method: classified into functional end-to-end anastomosis, overlapping anastomosis, delta anastomosis, and others. Additionally classified as intracorporeal or via a small laparotomy.
(17)  Methods for dissecting the right accessory colonic vein: dissection from the cranial and caudal sides.
(18)  Direction of lymph node dissection: the dissection procedure of the surgical trunk is classified into caudal to cranial, cranial to caudal, and caudal to cranial after setting a cranial termination point.
(19)  Lymph node dissection: classified as D0–1, D2, D3v, and D3a. The definitions are as follows.
  a For the ileocecal and right colic artery
    D2, the ileocecal or right colic artery is dissected proximally without SMV exposure; D3v, the anterior face of the SMV is exposed, and the ileocecal or right colic artery is dissected proximally; D3a, the anterior face of the SMA is additionally exposed, and the ileocecal or right colic artery is dissected at the root.
  b For the middle colic artery
    D2, the right/left branch of the middle colic artery (MCA) is dissected proximally. The main trunk of the MCA is not exposed; D3v, the main trunk of the MCA and anterior face of the SMV are exposed, and the right branch or trunk of the MCA is dissected proximally; D3a, the MCA main trunk and anterior face of the SMA are exposed, and the right branch or trunk of the MCA is dissected at the root.

The degree of dissection is judged based on the dissection of the dominant main vessel, regardless of SMV exposure during the procedure.

(20)  Synchronous other-organ resection: description of synchronously resected organs: duodenum, jejunoileum, colon, liver, gallbladder, pancreas, iliopsoas muscle, inferior vena cava, aorta, iliac artery and vein, SMA and SMV, peritoneum of the abdominal wall adjacent to the colon, gonadal vessels, mesentery of other intestinal tracts, and others.
(21)  Devices used during lymph node dissection: monopolar, bipolar (including sealing devices), ultrasonic coagulation, and incision devices.
(22)  Intraoperative vascular injury: intraoperative injury to any of the following vascular organs: SMA, SMV, accessory right colonic vein, ileocecal artery, right colonic artery, MCA, anterior superior pancreaticoduodenal vein, right gastroduodenal vein, gastrocolic trunk, terminal ileal branch, first jejunal vein, and others (e.g., inferior vena cava).

Injury is classified as:

(i) Haemostasis achieved by compression, application of a haemostatic agent, and cauterisation (including soft coagulation with suction), with the injured vessel preserved without transection; (ii) haemostasis with sutures (including the final addition of sutures after temporal haemostasis) to preserve the injured vessels; (iii) ligature of the injured vessel, ligature/clip of the injured vessel (including those added after temporal haemostasis), and vessel dissection (including those dissected for lymph node dissection); (iv) haemostasis achieved by compression, application of a haemostatic agent, or cauterisation (including soft coagulation with suction) of the injured vessel; however, the vessel is later dissected for lymph node dissection (if unsure between iii and iv, select iii); and (v) details will be provided for other cases (e.g. the injured vessel is cut, but haemostasis is achieved only by cauterisation and left alone, or the vessel is dissected, and haemostasis is achieved using an ultrasonic coagulation incision device).

(23)  Final bleeding control: possible/impossible (operative death).
(24)  Timing of intraoperative vascular injury: during lymph node dissection, mobilisation, intracorporeal anastomosis, extracorporeal anastomosis, and others (described at the time of adhesion dissection, drain insertion, etc.).
(25)  Other intraoperative complications: list those requiring intraoperative intervention (e.g., injury to other organs).
(26)  Open conversion: a skin incision ≥ 8 cm is generally defined as an open conversion. However, if the incision exceeds 8 cm solely for the purpose of tumor extraction, it shall not be considered an open conversion.
(27)  Postoperative complications (within 30 days): Clavien–Dindo classification grade ≥ II should be used.
(28)  Date of onset of anastomotic leakage/deep SSI: the date of onset is defined as the earliest time of abnormality during the initial physical examination, such as fever, abdominal pain, abnormal discharge from the drainage tube, blood test, and imaging.
(29)  Treatment intervention date: the date of treatment intervention is defined as the date corresponding to any of the following: administration of medical therapy (in the case of Clavien–Dindo classification grade II); performance of endoscopic, surgical, or interventional radiology (IVR) procedures without general anesthesia (grade IIIa); performance of endoscopic, surgical, or IVR procedures with general anesthesia (grade IIIb); or admission to the intensive care unit (ICU) (grade IV). Additionally, in cases of anastomotic leakage or deep surgical site infection (SSI), the date should be recorded if a new percutaneous drain is inserted or an existing one is replaced.
(30)  30-day death: death from any cause within 30 days of surgery.
(31)  Surgical death: 30-day death or death during hospitalisation 31–90 days postoperatively.
(32) Histological type, pathological stage, T and N stages, and R status: according to the Japanese Classification of Colorectal, Appendiceal, and Anal Carcinoma, third English Edition [37].
(33)  Postoperative adjuvant therapy: indicate whether at least one course has been completed, and specify the type of chemotherapy administered.
(34)  Recurrence-free survival: duration from the date of first treatment (surgery or start of preoperative therapy) to the date of the last confirmed recurrence-free/recurrence.
(35)  Local recurrence: local recurrence will be assessed based solely on the initial recurrence, with distinctions made between lymph node recurrence, anastomotic site recurrence, and other locations.
(36)  Intestinal obstruction during follow-up (Clavien–Dindo classification grade ≥ II): only the first occurrence 30 days after surgery will be considered.
(37)  Abdominal wall scar hernia: this will be diagnosed based on imaging findings, such as CT, or palpation of the hernia in the surgical scar. Only the first occurrence 30 days after surgery will be considered.

### Target number of cases

The target number of cases for the study is 2,000; nevertheless, we aim to recruit as many cases as possible within the study period.

The rationale for setting the target number of cases is described below.

The postoperative complication rate (grade ≥ III) of laparoscopic surgery in all patients with colon cancer in a Japanese randomised controlled trial (RCT) (JCOG0404) was 4.6% [3]. The postoperative complication rate (grade ≥ III) for laparoscopic CME of right colon cancer in a Norwegian RCT was 6.1% [10]. Based on these reports, we calculated that a sample size of approximately 2,000 cases is required to achieve a postoperative complication rate (grade ≥ III) of 5% and a 95% confidence interval within ± 1.0% based on the binomial distribution in this study.

### Statistical analysis

The analysis of primary endpoints, key secondary endpoints, and other secondary endpoints regarding short-term outcomes will begin as soon as 120 days after the patient enrolment period ends. Long-term outcome analysis of the secondary endpoint shall be performed upon completion of the 5-year follow-up period for all enrolled subjects.

Primary/key secondary endpoints: Summary statistics of patient background and surgical outcomes will be calculated, and the proportion of major complications and intraoperative vascular injury around the SMV and their causes will be summarised. Logistic regression analysis will be performed using the factors listed in the survey items that may influence the rate of major complications and intraoperative vascular injury around the SMV. A random-effects model will be used to account for the differences between institutions. Patients who underwent open conversion before the lymph node dissection will be excluded from the analysis of key secondary endpoints, and the relationship between the extent and location of the damaged vessel and the magnitude of the effect on the perioperative period will be considered.

Secondary endpoints: Summary statistics for patient background and other surgical outcomes will be calculated. A multivariate logistic regression or multiple regression analysis will be performed using the factors listed in the survey items that may influence outcomes. A random-effects model will be used to account for differences between the centres. The main endpoint for short-term outcomes for each subgroup is postoperative complications (Clavien–Dindo classification grade ≥ II), and group comparisons will be conducted after propensity score matching (differences between groups will be tested using Student’s t-test and the χ^2^ test) and logistic regression to identify confounders. Multiple regression analysis will be used to analyse continuous variables. A random-effects model will be used to account for the differences between institutions. The Kaplan–Meier method will be used to plot cumulative late complication curves, local and overall recurrence-free survival curves, and overall survival curves for long-term outcomes. To account for confounding factors, a multivariate Cox regression analysis will be performed using preoperative and postoperative factors that may contribute to recurrence and late complications, and risk factors will be examined. In addition to regression analysis of factors, each factor will be discussed from clinical and medical perspectives to reach a combined perspective.

The number of experienced cases will be added to the factors in the multivariate analysis to examine their effect on the outcome. The correlation of the number of cases experienced and outcomes (operation time and blood loss) with the learning curve will be evaluated using the moving average method and the cumulative sum curve.

Statistical significance will be set at *p* < 0.05.

## Discussion

Successfully performing surgical procedures without intra- or postoperative complications is critical. A national survey in Japan demonstrated that the mortality rate of right hemicolectomy was higher than that of low anterior resection [7]. However, no detailed studies regarding the factors influencing the safety of this procedure have been conducted. Therefore, we will conduct the SCaRLET study to identify the rate and cause of severe perioperative complications in laparoscopic right-sided colectomy.

To the best of our knowledge, this will be the first large prospective study of laparoscopic right-sided colectomy in Japan, with nearly equal numbers of laparoscopic and robotic procedures. This study is characterised by a low event selection bias owing to its prospective nature. It will be conducted at Japanese institutions that excel in JSLCS, with high proficiency levels of performing laparoscopic and robotic surgeries. Hence, the institutional experience will not affect the study outcomes, potentially determining the actual safety of the procedure. We will investigate the actual incidence and causes of high mortality rates due to the procedure, which have not yet been clarified. Furthermore, we will investigate the actual incidence and causes of intraoperative vascular injury, which may be the only critical aspect in D3 lymph node dissection.

A major operational challenge is the difficulty of case accumulation inherent in large-scale prospective registration. Although more than 70 institutions are participating nationwide, the success of the study depends on their consistent and proactive cooperation. Timely and accurate data entry, strict protocol adherence, and sustained engagement throughout the study period require substantial coordination. Although not addressed in other sections, these logistical demands are essential to the feasibility and execution of the study. Several strategies may be considered to address this operational challenge. Firstly, obtaining informed consent via the opt-out method, in accordance with the guidelines of the Regional Bureau of Health and Welfare, simplifies the consent process and may substantially lower the barrier to patient enrolment. This procedural efficiency is particularly beneficial in busy clinical settings, where it can contribute to improved recruitment rates. Secondly, sustained engagement of participating institutions may be promoted through regular progress updates and personalised communication. Periodic email distributions, online meetings, and study group sessions that emphasise the scientific significance and clinical relevance of the research may foster a sense of ownership and motivation among site investigators. Fortunately, the study topic currently attracts considerable attention nationwide, and continued outreach efforts will be made to maintain interest. Thirdly, detailed centralised support provided by the study office—such as a coordinating centre or help desk—can assist institutions with protocol compliance, data entry, and technical troubleshooting. This support system not only enhances data quality but also alleviates administrative burden at each site. Lastly, clearly recognising institutional contributions through authorship opportunities is expected to encourage active participation and reinforce accountability. In addition to the primary analysis, numerous secondary analysis themes are planned, thereby increasing opportunities for authorship. Investigators who meet authorship criteria will be credited as part of a group collaborative. Collectively, these measures are expected to mitigate logistical barriers and enhance the feasibility and execution of large-scale prospective studies.

Another concern is that cases with poor general condition may be excluded or that surgeons may adopt more conservative approaches to prioritise safety, potentially reducing the observed complication rates. And it is essential that all eligible cases be registered without modification, and that surgical procedures be performed strictly in accordance with routine clinical practice, without deviation. To ensure the validity and generalizability of the study findings, comprehensive data collection on patient demographics, surgical techniques, and perioperative variables is planned, thereby enabling precise interpretation of the clinical and procedural contexts in which the outcomes are generated.

Given the absence of prior nationwide studies, the SCaRLET study holds significant value not only within Japan but also in the broader global context. As a multicentre, prospective investigation conducted under standardised conditions across high-performing institutions, it may serve as a landmark for best practices domestically and globally. The findings have the potential to contribute to international surgical literature and guide the evolution of safe and effective approaches to right-sided colectomy worldwide.

In conclusion, this large-scale, nationwide, multicentre, prospective study aims to identify key factors affecting the safety of laparoscopic right-sided colectomy. The findings will provide actionable insights to enhance surgical safety and inform best practices in colorectal surgery across Japan and worldwide.

## Supplementary Information

Supplementary Material 1. Rationale for the definitions of the approaches.

## Data Availability

The datasets used and/or analysed during the current study will be available from the corresponding author on reasonable request.

## Abbreviations

CME: Complete mesenteric excision
CT: Computed tomography
CVL: Central vascular ligation
ESSQS: Endoscopic Surgical Skill Qualification System
IVR: Interventional radiology
JSLCS: Japan Society of Laparoscopic Colorectal Surgery
MCA: Middle colic artery
RCT: Randomised controlled trial
SMA: Superior mesenteric artery
SMV: Superior mesenteric vein
SSI: Surgical site infection
TAPP: Transabdominal preperitoneal
