# Supplementary material for: Prospective observational study investigating the factors impacting the safety of endoscopic right-sided colectomy for colon cancer in Japan (SCaRLET study): a study protocol
Source: BMC Surg. 2025 Nov 29;26:3. doi: 10.1186/s12893-025-03362-1 (PMC12764104; doi:10.1186/s12893-025-03362-1)
Supplement: Supplementary file 1 — Supplementary Material 1. Rationale for the definitions of the approaches. [file 12893_2025_3362_MOESM1_ESM.docx]

**Supplementary Figure 1a**

**
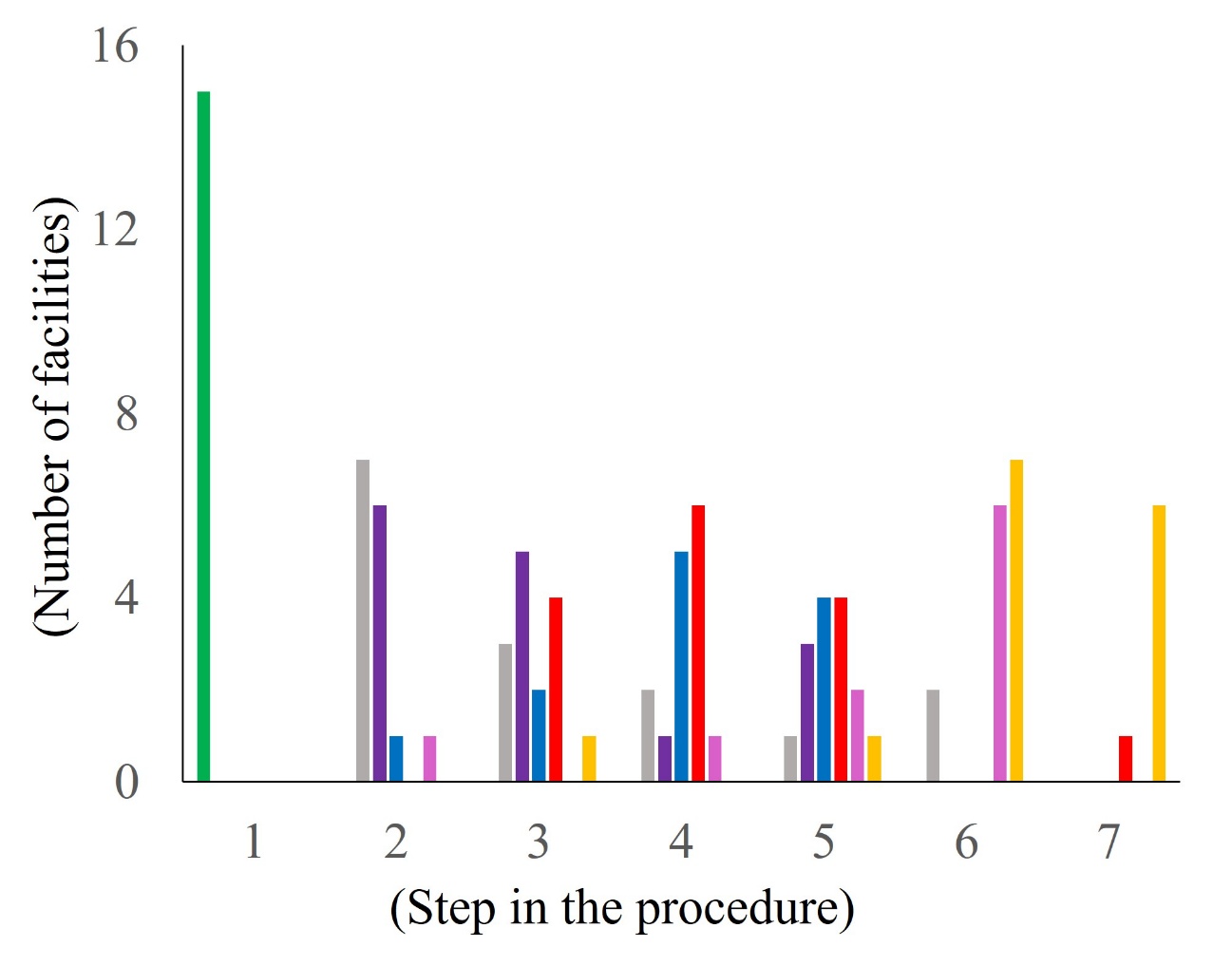
**

The order of each step in Cranial approach in laparoscopic surgery

Procedures vary among facilities. However, procedures in all facilities fit within the definition.

Green bar indicates opening of the omental bursa; gray bar, dissection of hepatic attachment; purple bar, dissection of the accessory right colic vein; red bar, node dissection of the surgical trunk; blue bar, dissection of the ileocecal artery and vein from the retroperitoneum; pink bar, mobilisation of the caudal attachments; yellow bar, mobilisation of the lateral attachments.

**Supplementary Figure 1b**

**
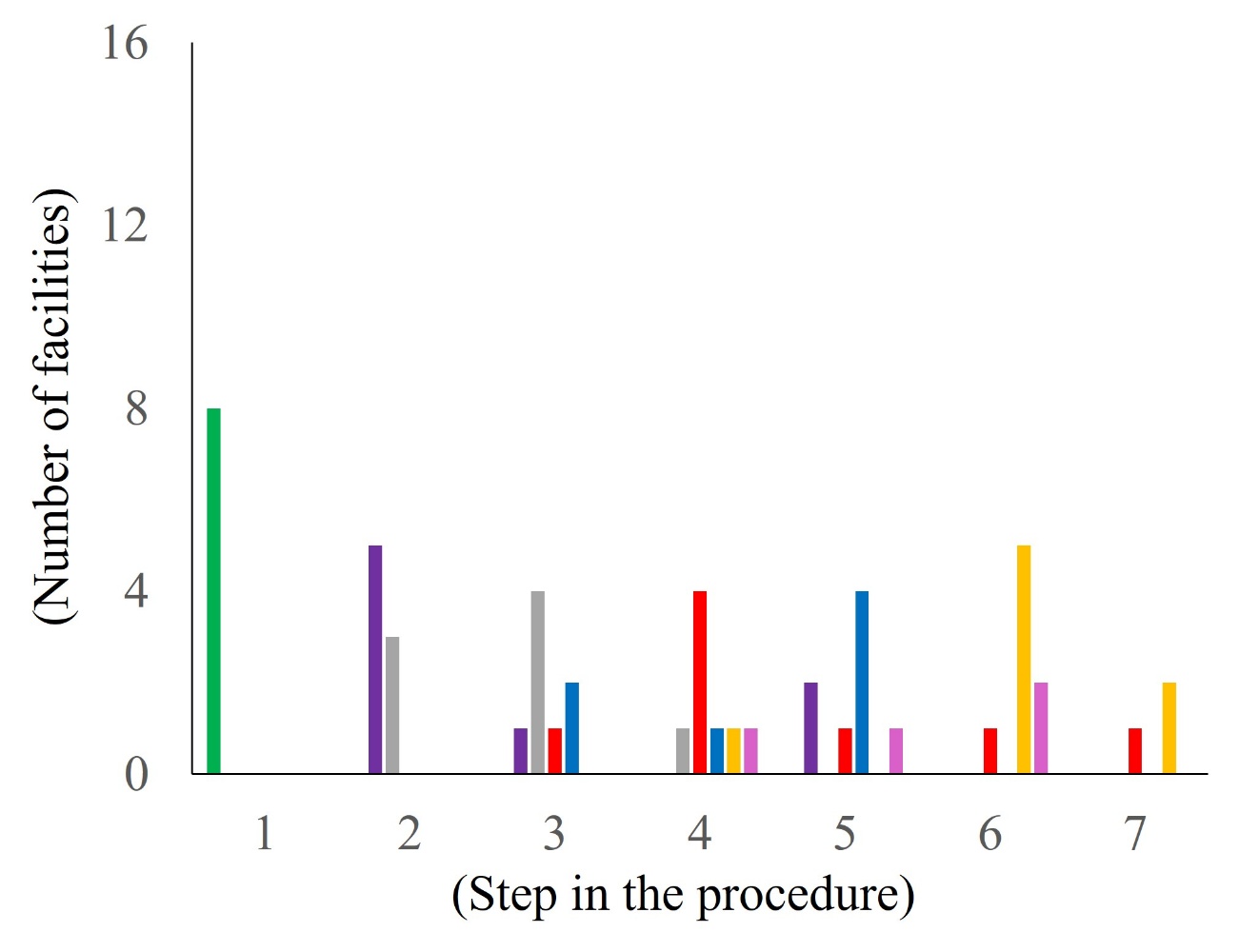
**

The order of each step in Cranial approach in robotic surgery

Procedures vary among facilities. However, procedures in all facilities fit within the definition.

Green bar indicates opening of the omental bursa; purple bar, dissection of the accessory right colic vein; gray bar, dissection of hepatic attachment; red bar, node dissection of the surgical trunk; blue bar, dissection of the ileocecal artery and vein from the retroperitoneum; pink bar, mobilisation of the caudal attachments; yellow bar, mobilisation of the lateral attachments.

**Supplementary Figure 2a**

**
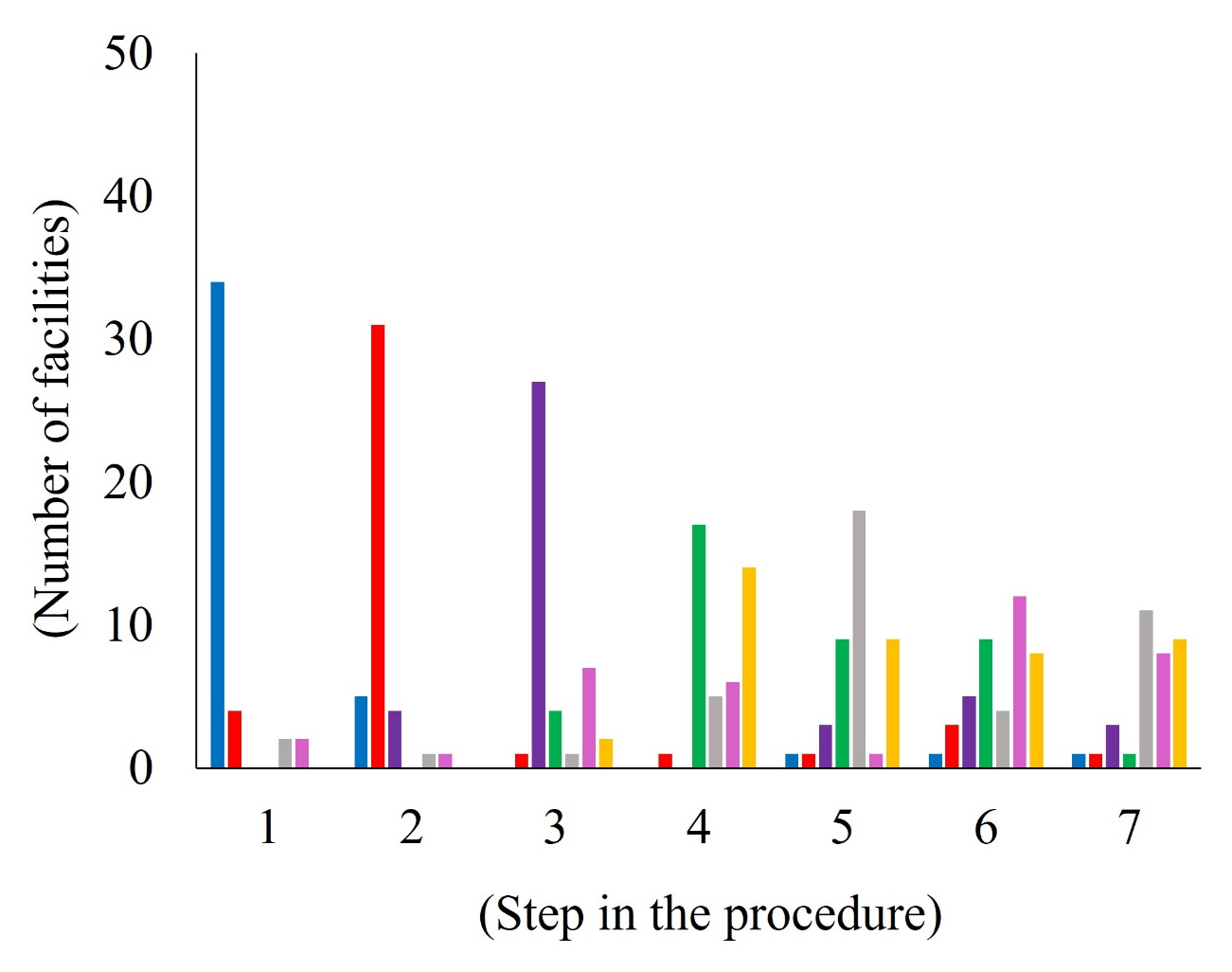
**

The order of each step in Medial approach in laparoscopic surgery

Procedures vary among facilities. However, procedures in all facilities fit within the definition.

Blue bar indicates dissection of the ileocecal artery and vein from the retroperitoneum; red bar, node dissection of the surgical trunk; purple bar, dissection of the accessory right colic vein; green bar, opening of the omental bursa; gray bar, dissection of hepatic attachment; pink bar, mobilisation of the caudal attachments; yellow bar, mobilisation of the lateral attachments.

**Supplementary Figure 2b**

**
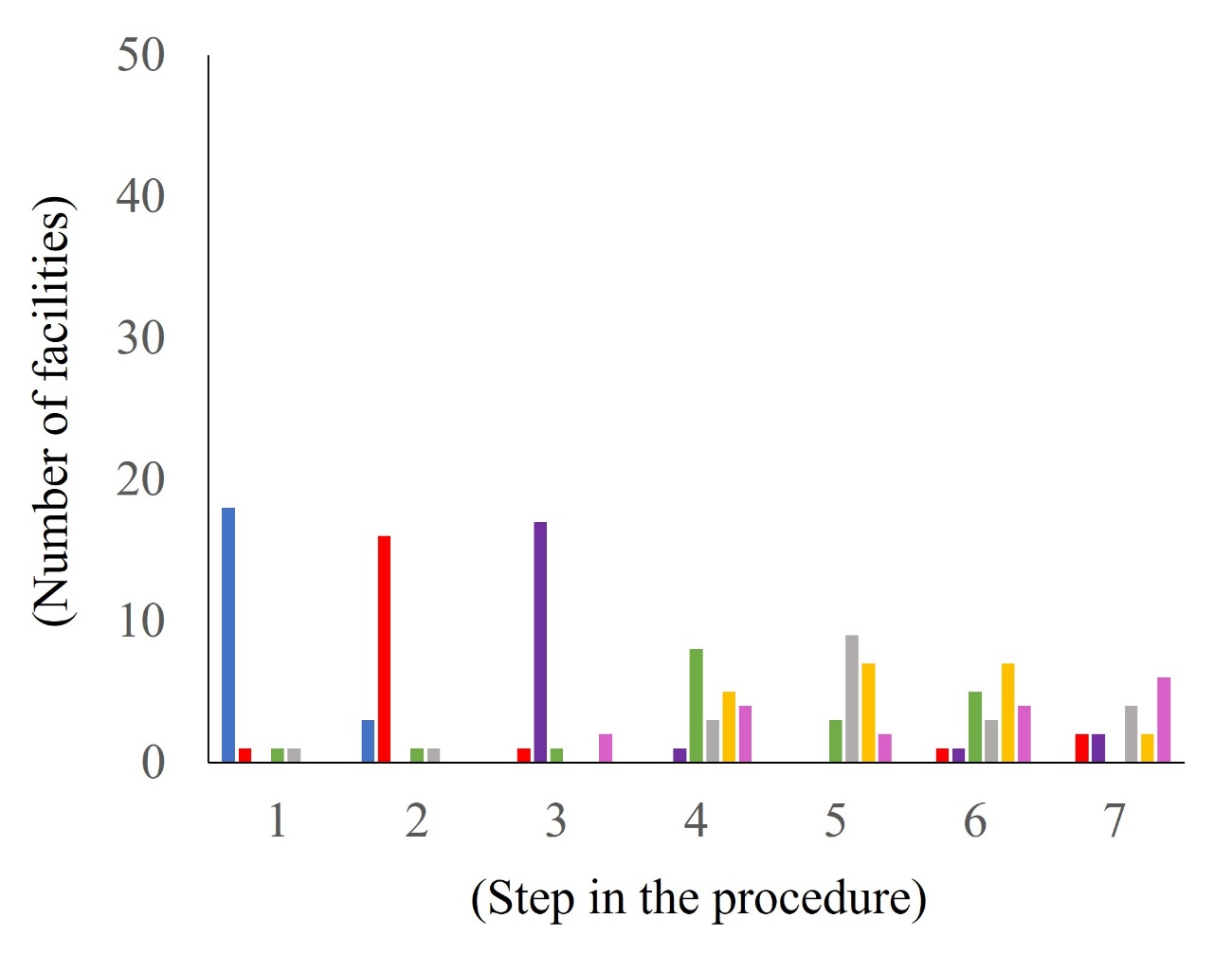
**

The order of each step in Medial approach in robotic surgery

Procedures vary among facilities. However, procedures in all facilities fit within the definition.

Blue bar indicates dissection of the ileocecal artery and vein from the retroperitoneum; red bar, node dissection of the surgical trunk; purple bar, dissection of the accessory right colic vein; green bar, opening of the omental bursa; gray bar, dissection of hepatic attachment; pink bar, mobilisation of the caudal attachments; yellow bar, mobilisation of the lateral attachments.

**Supplementary Figure 3a**

**
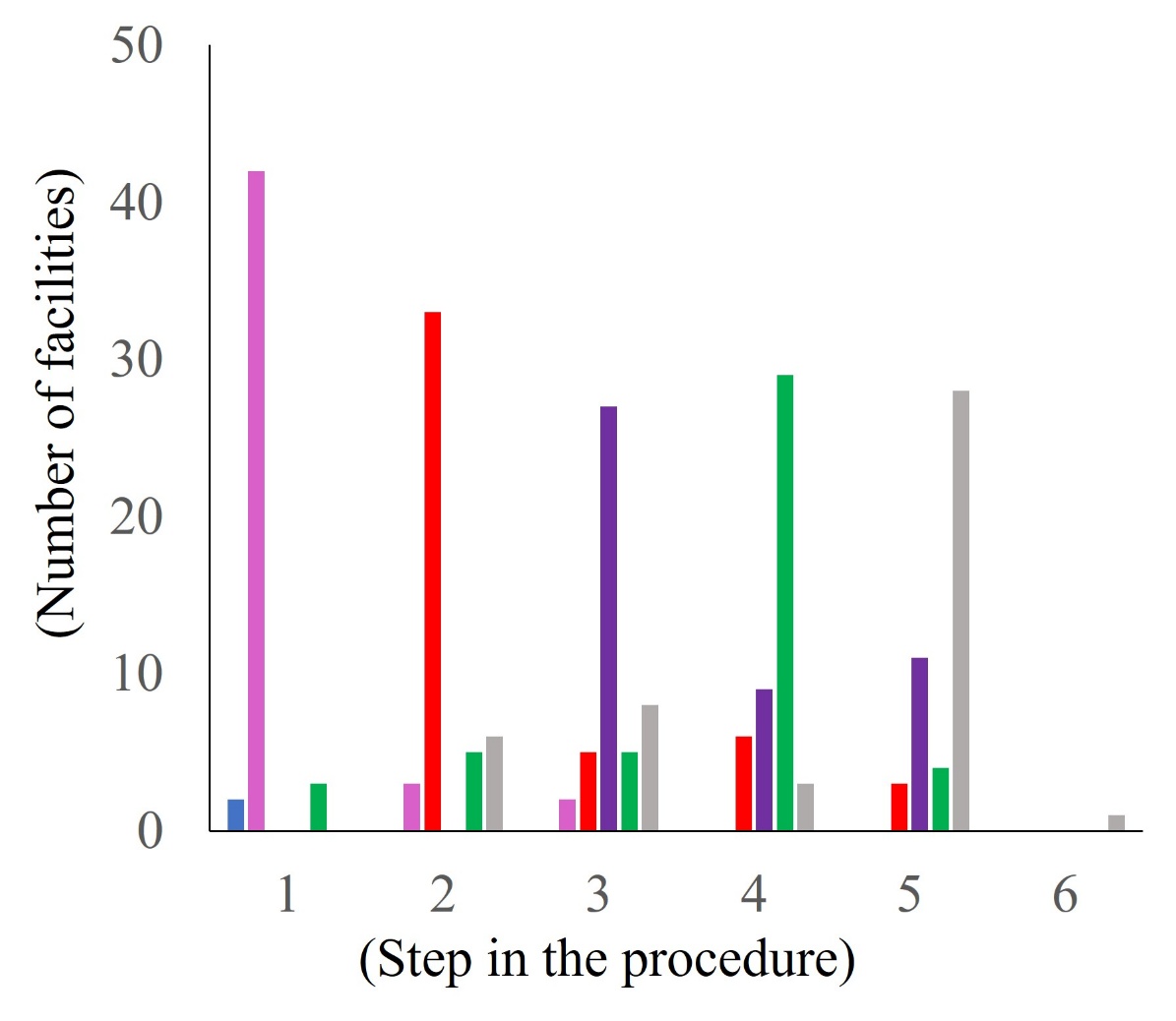
**

The order of each step in Retroperitoneal approach in laparoscopic surgery

Procedures vary among facilities. However, procedures in all facilities fit within the definition.

Pink bar indicates mobilisation of the caudal attachments; red bar, node dissection of the surgical trunk; purple bar, dissection of the accessory right colic vein; green bar, opening of the omental bursa; gray bar, dissection of hepatic attachment; blue bar, dissection of the ileocecal artery and vein from the retroperitoneum.

**Supplementary Figure 3b**

**
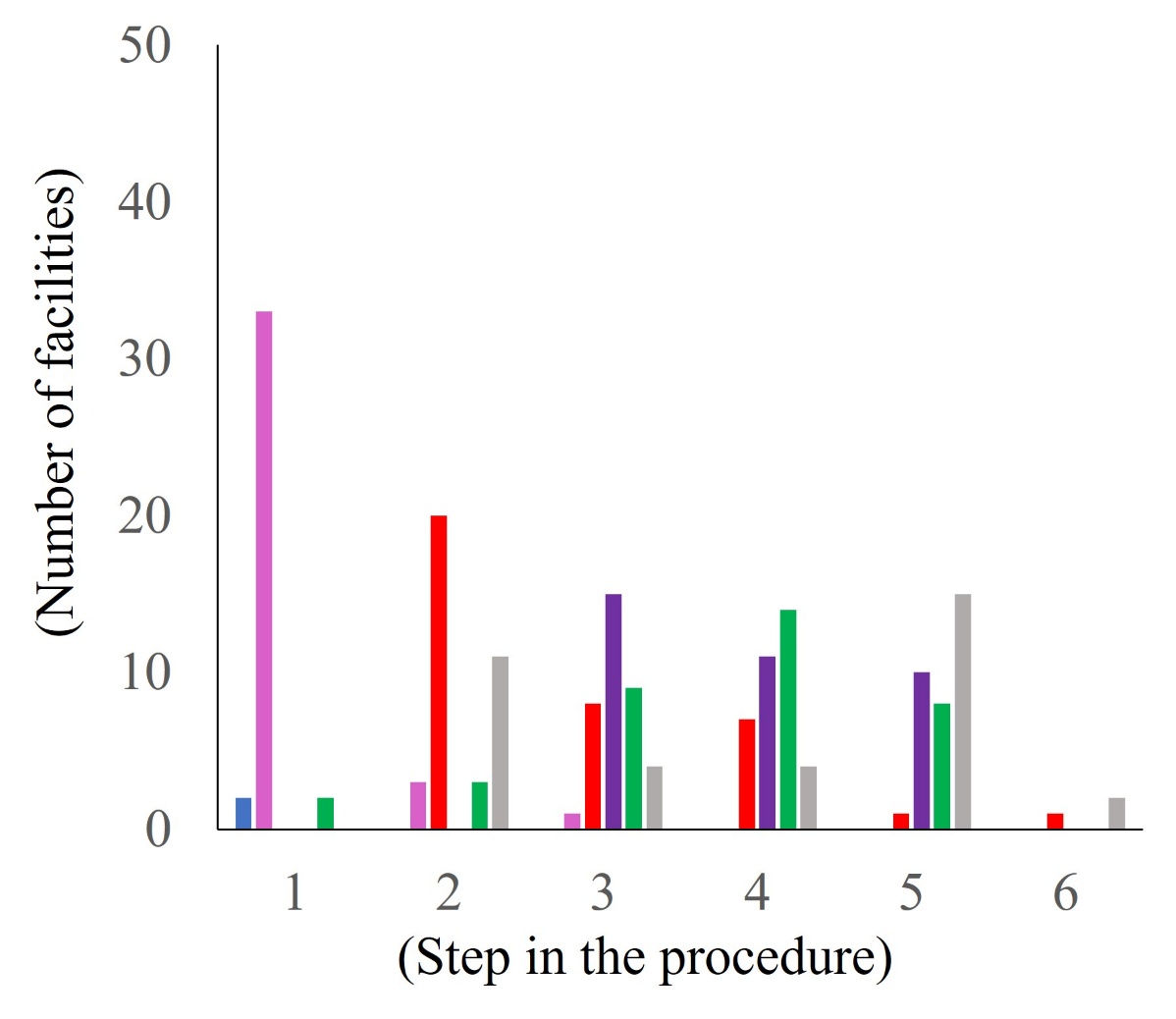
**

The order of each step in Retroperitoneal approach in robotic surgery

Procedures vary among facilities. However, procedures in all facilities fit within the definition.

Pink bar indicates mobilisation of the caudal attachments; red bar, node dissection of the surgical trunk; purple bar, dissection of the accessory right colic vein; green bar, opening of the omental bursa; gray bar, dissection of hepatic attachment; blue bar, dissection of the ileocecal artery and vein from the retroperitoneum.
